# Molecular insights into the historic demography of bowhead whales: understanding the evolutionary basis of contemporary management practices

**DOI:** 10.1002/ece3.374

**Published:** 2013-01-10

**Authors:** C D Phillips, J I Hoffman, J C George, R S Suydam, R M Huebinger, J C Patton, J W Bickham

**Affiliations:** 1 Department of Biological Sciences, Texas Tech UniversityLubbock, Texas; 2 Department of Animal Behaviour, University of BielefeldBielefeld, North Rhine-Westphalia, Germany; 3 Department of Wildlife Management, North Slope BoroughBarrow, Alaska; 4 Southwestern Medical Center, University of TexasDallas, Texas; 5 Department of Forestry and Natural Resources, Purdue UniversityWest Lafayette, Indiana; 6 Battelle Memorial InstituteHouston, Texas

**Keywords:** Approximate Bayesian computation, bottleneck, bowhead whale, generation time, historic demography, mutation rate.

## Abstract

Patterns of genetic variation observed within species reflect evolutionary histories that include signatures of past demography. Understanding the demographic component of species' history is fundamental to informed management because changes in effective population size affect response to environmental change and evolvability, the strength of genetic drift, and maintenance of genetic variability. Species experiencing anthropogenic population reductions provide valuable case studies for understanding the genetic response to demographic change because historic changes in the census size are often well documented. A classic example is the bowhead whale, *Balaena mysticetus*, which experienced dramatic population depletion due to commercial whaling in the late 19th and early 20th centuries. Consequently, we analyzed a large multi-marker dataset of bowhead whales using a variety of analytical methods, including extended Bayesian skyline analysis and approximate Bayesian computation, to characterize genetic signatures of both ancient and contemporary demographic histories. No genetic signature of recent population depletion was recovered through any analysis incorporating realistic mutation assumptions, probably due to the combined influences of long generation time, short bottleneck duration, and the magnitude of population depletion. In contrast, a robust signal of population expansion was detected around 70,000 years ago, followed by a population decline around 15,000 years ago. The timing of these events coincides to a historic glacial period and the onset of warming at the end of the last glacial maximum, respectively. By implication, climate driven long-term variation in Arctic Ocean productivity, rather than recent anthropogenic disturbance, appears to have been the primary driver of historic bowhead whale demography.

## Introduction

Inference on the demographic history of species is an important component of understanding how evolutionary processes shape contemporary patterns of genetic variation (Waples 2005). The measurable genetic signatures of demographic change are a manifestation of a combination of effects relating to the timing, extremity, and complexity of demographic history. Demographic events must be of sufficient magnitude to produce a statistically detectable genetic effect, and event procession in a dynamic demographic history can erode signal for ancestral events (Johnson et al. 2007; Listman et al. 2007; Heled and Drummond 2008). These parameters weigh heavily on historic demographic inference, yet additional important considerations include the resolving capabilities of different types of genetic marker used for inference (which are in turn dictated by mutation rates, ploidy, mode of inheritance, variability, and the number of loci) and the analytical methods available to interrogate the data. For example, Hoffman et al. (2011) recently demonstrated differences in the utility of mitochondrial sequence and microsatellite data for reconstructing the demographic history of Antarctic fur seals (*Arctocephalus gazella*) under an approximate Bayesian computation framework. Apart from the above considerations, the frequent absence of strong a priori hypotheses about species' demographic history can occlude model development and result validation. For this reason, species for which strong a priori demographic information are available can provide useful case studies for identifying factors that underly demographic change over multiple timescales.

Several analytical approaches have been developed to investigate signal for historic demographic change from genetic data. Some of these, developed for sequence data, include commonly applied statistics such as Tajima's *D* (Tajima 1989), Fu's *F*_*S*_ (Fu 1997), and the raggedness index (Harpending 1994). These methods rely on the comparison of average pairwise distances and segregating sites (as *θ* estimators), allelic diversity, and the frequency distribution of pairwise distances, respectively. The first two of these methods are classically defined as neutrality tests, although demographic changes are also readily detected, and all three can be influenced by factors other than demography such as selection and migration (Tajima 1989; Harpending 1994; Fu 1997). Statistical significance rendered through Tajima's *D*, Fu's *F*_*S*_, and the raggedness index all require some number of generations to have passed for new mutations or genetic drift to produce a measurable effect. The timing of signified events cannot be determined using the first two methods, so must be postulated from external data. For population expansions detected through the raggedness index (but not population bottlenecks), timing of the event can be inferred by making assumptions about *μ* and generation time. An alternative approach, also developed for sequence data, Bayesian skyline analysis (Drummond and Rambaut 2007), reconstructs demographic changes over time using the coalescent and directly from a sequence alignment and the estimated phylogeny. As genetic signal for demography at any given time is related to the genetic variability at that phylogenetic depth, this approach uniquely provides an estimated continuous demographic reconstruction over time without the need for a priori demographic assumptions.

Other tests capable of detecting demographic change, explicitly defined as “bottleneck analyses”, exploit expected changes in microsatellite distributions resulting from genetic bottlenecks, including changes in allele frequency, heterozygosity, or allele size distributions. Probably, the most well-known and widely applied of these is the heterozygosity excess test, developed by Luikart et al. (1998), which detects signal for the expected transient excess of heterozygosity that can arise during genetic bottlenecks. The heterozygosity excess test relies on a transient phenomenon (expected to last 4 × *N*_*e*_ generations, where *N*_*e*_ is the bottleneck population size), that dissipates as genetic drift and new mutations re-establish mutation-drift equilibrium (Luikart et al. 1998 and references therein). A very different approach that can be applied to understanding demographic change using microsatellite and/or sequence data is approximate Bayesian computation (ABC, Beaumont et al. 2002). This method employs coalescent-based simulations of alternative demographic scenarios, which are compared to the observed data through summary statistics. Simulated data producing summary statistics most similar to the observed are retained to estimate the posterior distributions of demographic parameters, such as the timing of events, associated effective population sizes (*N*_*e*_), and marker mutation rates (*μ*). ABC provides the flexibility to explore genetic signals over time frames specified a priori, but poor fit to the model can indicate the influence of an unaccounted history. An important caveat of this method is the reliance, to a certain extent, on a priori information that is used to parameterize simulations. This reliance has been considered a limiter of new discovery (Templeton 2009), yet ABC has also been considered valuable for developing strong demographic inference when thoroughly implemented (Nielsen and Beaumont 2009; Csilléry et al. 2010). ABC has proven powerful for understanding bottleneck effects in species with exceptionally well-characterized recent demographic reductions (Chan et al. 2006; Hoffman et al. 2011) among other demographic scenarios (Estoup et al. 2004; Fagundes et al. 2007).

Many species of marine mammals, especially whales, are characterized by recent histories involving drastic population reduction due to unregulated commercial harvesting. As such human-induced population declines are often well documented in terms of duration and severity, demographic studies in these species are often well parameterized, providing clear a priori expectations, at least for recent events. Furthermore, whales possess a unique combination of life-history characteristics, including long generation times, a parameter that is important in determining the genetic response to population reduction (Allendorf 1986). Baleen whales are also unique in both their large body size and dietary strategy, relying on planktonic communities as a food source, which vary in abundance according to ocean productivity. Productivity dictates the carrying capacity of marine ecosystems, and is itself largely driven by temperature (Behrenfeld et al. 2006). Consequently, historic demographic changes in baleen filter feeding species could potentially mirror long-term climactic variations. From a practical standpoint, general public interest and continued aboriginal, scientific, and commercial harvest of some whale species also provide increasing relevance to demographic studies of whales.

**Figure 1:**
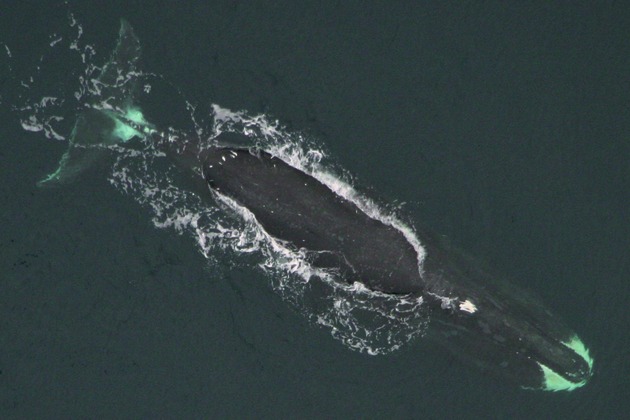
Photograph of a bowhead whale taken near Point Barrow, Alaska in 2005 (photographer: J. C. George, Scientific Research Permit 782–1719). Note the whitish markings on the peduncle and scars on the back. Both are indications of advancing age. The maximum age of bowhead whales has been estimated at ∼200 year with an average age at sexual maturity >20 years.

The bowhead whale, *Balaena mysticetus*, is the second largest species of animal, has an estimated generation time of more than 50 years (Taylor et al. 2007), and may routinely live for over a 100 years, with a maximum age possibly exceeding 200 years (Fig. 1; George et al. 1999; Rosa et al. 2011). Bowheads are an important food source for some native communities along the coast of NW Alaska and E Chukotka. These communities take part in an annual subsistence harvest under a quota system regulated through a co-management agreement by NOAA and the Alaska Eskimo Whaling Commission and the International Whaling Commission (Suydam et al. 2010). There are five recognized stocks of bowhead whales, which are characterized by differences in geographic distribution, migration patterns, and demographic trends (Alter et al. in press). Of these, the Bering-Chukchi-Beaufort Seas stock (BCB) is the largest and currently most intensely harvested. In relation to the BCB, genetic investigations have demonstrated that while the Okhotsk and Atlantic stocks are isolated and significantly differentiated sharing little migration with the BCB, connectivity between the BCB and the Canadian stock is considerably higher (Bickham et al. 2009; Givens et al. 2010). Within the BCB population structure among localities has been thoroughly investigated through which support for this hypothesis was lacking (Givens et al. 2010). Observation and sampling of individuals at localities through annual harvests occurs during seasonal migratory events. Previous studies have investigated differences in the relatedness of groups of migratory individuals throughout spring and autumn migration (Jorde et al. 2007; Givens et al. 2010). One of these studies (Jorde et al. 2007) found elevated genetic differentiation between whales collect about a week apart. However, Givens et al. (2010), using a panel of 22 microsatellites developed specifically for bowhead whales, did not recover this pattern, and concluded after careful analysis that this pattern was confined to the separate set of 10 loci used by Jorde et al. (2007). Thus, the BCB population is currently managed as a single stock.

Records of past events and previous scientific investigations suggest that the BCB may have experienced a dynamic demographic history. Unregulated commercial whaling from 1848 to 1914 killed a total of 18,684 bowheads and resulted in an estimated 93% population reduction in the BCB, reducing the population to an estimated 1000 individuals (Woodby and Botkin 1993; Rooney et al. 2001; Punt 2006). Koski et al. (2010) estimated the contemporary census size of the BCB (in 2004) to be 12,631 and, George and Zeh (2012) estimated the current rate of population increase to be 3.5% annually. No studies have been able to realize a genetic signal for the anthropogenic population depletion (Rooney et al. 1999; Hunter 2005; Givens et al. 2010). Through investigation of more ancient demographic phenomena, Rooney et al. (1999) reported signal for a population expansion estimated to have occurred 8500 years before present (ybp). These authors note that the timing of this proposed population expansion is coincident with the formation of the M'Clintock Channel Sea Ice Plug, which would have isolated the BCB from the eastern Canadian Arctic stock (Dyke et al. 1996).

This study uses information about harvest-induced population reduction, contemporary census population size increase, and climatic chronology to explore the factors determining demographic signal and how this signal can be detected through different analytical approaches. Autosomal microsatellites and mitochondrial sequence data are the two most widely applied marker types in demographic studies. For this study, a panel of 22 microsatellite loci specifically designed for *B. mysticetus* (Huebinger et al. 2008; Givens et al. 2010) and three mitochondrial gene regions (Cytb, NDI, and HVRI) are investigated. Specifically, we test the hypothesis for a recent bottleneck and explore historic demographic trends in relation to episodes of climate change. The results of this study provide insights into how life-history characteristics of *B. mysticetus* have been central in determining the genetic response to demographic change.

## Materials and Methods

### Sample collection and DNA extraction

Tissues were collected from 324 whales from the BCB stock of bowhead whales. All specimens were analyzed for 22 microsatellites and 168 were analyzed for three mitochondrial gene regions. A total of 305 individuals were previously genotyped by Givens et al. (2010) and the remainder was genotyped for this study. Control region sequences (HVRI) were previously reported by LeDuc et al. (2008) and the protein coding genes (Cytb and ND1) are reported here for the first time. Sequences are available at Genbank under accession numbers JX470203-JX470262. The majority of the samples were obtained via necropsy sampling of whales that were part of Alaskan aboriginal subsistence harvests. Six tissue samples were obtained from remote biopsy darting. Table 1 presents sampling locations (8 Alaskan villages where whales were landed) and number of samples collected from each location. Tissues and DNA samples were stored at −80°C. DNA was extracted from skin slices or biopsy plugs using the GenElute mammalian genomic DNA purification kit (Sigma-Aldrich; St. Louis, MO).

**Table 1:** Summary of number of samples and sampling location for each population for microsatellites and mitochondrial DNA (mtDNA) datasets

Location	N microsatellites	N mtDNA
Barrow	260	137
Gambell	9	4
Savoonga	19	10
Kaktovik	16	9
Little Diomede	1	0
Nuiqsut	5	3
Point Hope	7	1
Wainwright	7	4
Total	324	168

### Microsatellite genotyping

Genotypes for 22 microsatellite loci were generated as detailed in Huebinger et al. (2008) and Givens et al. (2010). The loci are described in Table 2. Allele sizes were determined by fragment separation on an ABI3100 DNA Analyzer (Applied Biosystems, Inc., Foster City, CA) using GeneScan-400 (ROX) size standard. Alleles were assigned in GeneMapper version 4.0 (Applied Biosystems, Inc.). Samples that produced poor quality chromatograms or failed to amplify were reanalyzed. A thorough description of the microsatellite dataset was previously reported by Givens et al. (2010).

**Table 2:** Details of the 22 microsatellite loci used in this study, including literature sources and polymorphism characteristics in 324 Bowhead whales. *P*-values significant at *α* < 0.05 without correction for multiple statistical tests are highlighted in bold

Locus	Number of alleles	Observed heterozygosity (H_O_)	Expected heterozygosity (H_E_)	Hardy–Weinberg equilibrium probability	Probability of homozygote excess	Null allele frequency
Bmy1_1	10	0.822	0.812	0.159	0.550	−0.007
Bmy2_1	11	0.756	0.774	0.474	0.163	0.011
Bmy7_1	12	0.811	0.791	0.354	0.069	−0.014
Bmy8_1	16	0.783	0.800	0.504	0.095	0.010
Bmy10_1	22	0.890	0.926	0.386	**0.022**	0.019
Bmy11_1	14	0.870	0.878	0.274	0.485	0.004
Bmy12_1	27	0.930	0.922	0.653	0.227	-0.005
Bmy14_1	6	0.503	0.551	0.172	**0.015**	0.045
Bmy16_1	8	0.803	0.770	0.404	0.542	−0.022
Bmy18_1	17	0.881	0.902	0.693	0.099	0.011
Bmy19_1	16	0.843	0.867	0.282	0.052	0.013
Bmy26_1	22	0.895	0.925	0.385	**0.028**	0.016
Bmy33_1	13	0.798	0.807	**0.007**	0.508	0.005
Bmy36_1	28	0.938	0.939	0.787	0.506	0.000
Bmy41_1	22	0.896	0.905	0.088	**0.035**	0.004
Bmy42_1	11	0.722	0.781	0.397	**0.033**	0.039
Bmy49_1	24	0.895	0.892	0.098	0.390	−0.003
Bmy53_1	17	0.881	0.877	0.388	0.070	−0.003
Bmy54_1	8	0.680	0.707	0.237	**0.022**	0.019
Bmy55_1	6	0.682	0.710	**0.005**	**0.040**	0.019
Bmy57_1	9	0.566	0.602	**0.006**	**0.000**	0.029
Bmy58_1	27	0.929	0.926	**0.032**	0.450	−0.002
Average	15.7	0.808	0.821	–	–	

### Mitochondrial DNA (mtDNA) sequencing

Sequences from three mitochondrial gene regions, HVRI (397 base pairs), Cytb (1140 base pairs), and ND1 (957 base pairs) were generated using PCR and Sanger sequencing protocols. Detailed laboratory methods are described in LeDuc et al. (2008) for HVRI and Phillips et al. (2011) for the two protein coding genes.

### Classical sequence-based demographic tests

Concatenation of the mitochondrial gene regions resulted in a 2494-base pair sequence for each individual. One-hundred-sixty-eight individuals sequenced for all three gene regions were included for analyses (Table 1). Arlequin version 3.5.1.2 (Excoffier et al. 2005) was used to calculate a variety of summary statistics. As initial descriptors of variability, number of haplotypes, number of variables sites, number of pairwise differences, and nucleotide diversity (Tajima 1983) were calculated. Tajima's *D,* useful for detecting departures from population equilibrium, selection, and rate heterogeneity, was calculated based on uncorrected pairwise comparisons. Significance was assessed by randomly generating samples under the hypothesis of neutrality and observing the proportion of simulated values less than or equal to the observed (Hudson 1990; Excoffier et al. 2005). Fu's *F*_*S*_, a test statistic that is sensitive to departure from population equilibrium, was also calculated. While Tajima's *D* relies on the comparison of estimators of *θ*, Fu's *F*_*S*_ relies on comparison to the observed allelic abundance to that obtained through simulation under neutrality. Similar to that implemented for Tajima's *D*, significance for Fu's *F*_*s*_ is obtained by the proportion of times simulated *F*_*S*_ values are equal to or smaller than the observed. A mismatch distribution and the associated raggedness index were also calculated. The mismatch distribution was based on uncorrected pairwise differences, and significance was based on the sum of squared deviations of the observed and expected mismatches that were obtained by generating 1000 random samples according to the estimated demography. Having recovered a non-significant raggedness index, the timing of the postulated demographic expansion was estimated assuming a generation time of 52 years (Taylor et al. 2007), an overall mutation rate (*μ*) of 6.14 × 10^−7^ per nucleotide per generation (obtained by averaging independent rate estimates for each gene region obtained through Bayesian demographic reconstructions, see below), and following the algorithms of Watterson (1975), Rogers (1995), and Schneider and Excoffier (1999) for estimating τ (τ = 2*μt*, where *t* is the time to demographic expansion).

### Extended Bayesian skyline plot

To explore signal contained in the mitochondrial dataset for demographic change over time, an extended Bayesian skyline plot (EBSP) was calculated. Bayesian skyline plots are based on the coalescent and use the estimated phylogeny to reconstruct demographic change without prior assumptions on the timing of events (Drummond and Rambaut 2007). The EBSP is an implementation of the Bayesian skyline plot that incorporates multi-locus information in the demographic reconstruction. As discussed by Heled and Drummond (2008), multi-locus perspectives about demographics empower reconstructions. Although the entire mitochondrial genome is functionally a single locus, EBSP was implemented to allow separate phylogenetic reconstructions for each mitochondrial gene using an HVRI mutation rate of 2.8%/million years (95% CI = 1.5–4.6%). This value was previously estimated by Bickham et al. (2009) following the methods of Alter and Palumbi (2009). ND1 and Cytb were assigned relaxed molecular clocks (as deemed appropriate through clock testing in MEGA version 5; Tamura et al. 2011) and their rates were estimated from the data in relation to that assumed for HVRI. Fifty-million MCMC sets were conducted, with sampling every 1000 iterations. Effective samplings of prior and posterior tree distributions were confirmed in Tracer (Rambaut and Drummond 2007). The last 10,000 iterations of simulations were retained for demographic reconstruction. Simulations and skyline plotting were repeated three times and inferred demographic trends were compared across analyses for consistency.

### Microsatellite data checking

Tests for deviation from Hardy–Weinberg equilibrium and for linkage disequilibrium were implemented using GENEPOP v. 3.1d (Raymond and Rousset 1995). Bonferroni adjustments (Hochberg 1988) with an *α* level of *P* ≤ 0.05 were carried out on all tabulated results. GENEPOP was also used to determine expected and observed heterozygosity (*H*_*E*_ and *H*_*O*_, respectively). Null allele frequencies were calculated following Chakraborty (1988) using the program Micro-checker (Van Oosterhout et al. 2004). Microsatellite error rates for the previoulsly published portion of this dataset were investigated by Morin et al. (2009).

### Classical microsatellite-based bottleneck tests

To test for evidence of a recent demographic decline or expansion, we analyzed the microsatellite data for deviations from expected heterozygosity at mutation-drift equilibrium within the program BOTTLENECK v 1.2.02 (Piry et al. 1999). Six different mutation models were evaluated: the strict Stepwise Mutation Model (SMM, Kimura and Ohta 1978), the Infinite Alleles Model (IAM, Kimura and Crow 1964), and four intermediate Two-Phase Models (TPMs) with 1%, 5%, 10%, and 30% IAM mutations, respectively. For each mutational model, the heterozygosity of each locus expected at equilibrium given the observed number of alleles (*H*_*eq*_) was determined using 10,000 simulations and then compared against observed heterozygosity (*H*_*e*_). We then recorded the number of loci for which *H*_*e*_ was greater than *H*_*eq*_ and smaller than *H*_*eq*_, and determined whether the overall set of deviations was statistically significant using sign, standardized differences, and Wilcoxon signed ranks tests. Finally, BOTTLENECK was also used to generate a qualitative descriptor of whether the observed allele frequencies at each locus deviate from the L-shaped distribution expected under mutation-drift equilibrium Luikart et al. (1998).

### Approximate Bayesian computation

**Figure 2:**
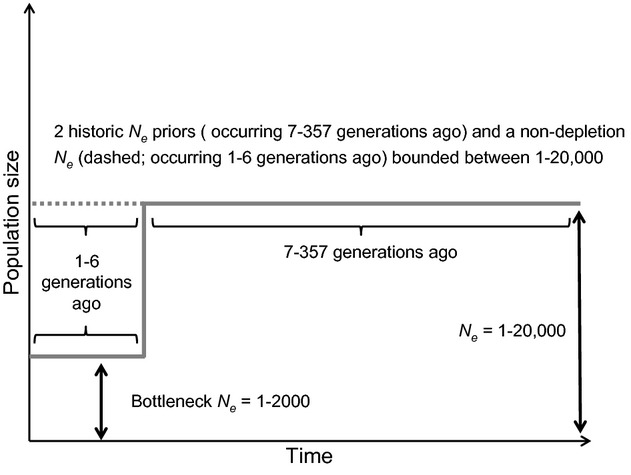
Models of demographic history of bowhead whales tested through ABC.

Approximate Bayesian computation (ABC), originally introduced by Beaumont et al. (2002), was implemented in DIYABC v1 (Cornuet et al. 2008, 2010). Demographic models were defined to capture any signal present in the genetic data specific to an anthropogenically induced population reduction. Information on the timing and severity of this reduction served as a priori demographic information around which models tested through ABC were developed. Underlying parameters to ABC analyses are generation time and sex ratio. Here, generation time was defined as 52 years, as reported by Taylor et al. (2007), and a 1:1 sex ratio was implemented based on data from Nerini et al. (1984), Heide-Jørgensen et al. (2010), and J. C. George (unpubl. data). Two demographic models were simulated for comparison, one being a model enforcing a population size reduction having priors for timing and severity bracketing known values, whereas in the second model, this population size reduction was not enforced. Models are graphically depicted in Figure 2 and summarized below. These models were defined by identical priors on time to account for historic events. Specifically, time priors employed represented a demographic event uniformly distributed between one and six generations ago (encompassing the timing of the known period of unregulated whaling), and two additional time priors uniformly distributed with equivalent prior distributions between 7 and 357 generations ago (population expansion, and ancestral population size). The upper bound on these time parameters extended to 18,500 ybp, liberally surrounding the timing of the postulated population expansion of Rooney et al. (1999). Prior distributions on *N*_*e*_ associated with each time window (and including the estimate of contemporary *N*_*e*_) were defined by uniform distributions between 1 and 20,000, with the exception of the bottleneck model, which incorporated a uniform *N*_*e*_ prior of 1–2000 associated with the initial time prior (1–6 generations). The purpose of confining the prior distribution on *N*_*e*_ at this time period was to enforce *N*_*e*_ values during this interval to correspond with knowledge of the census population size at that time. As such, demographic models were identical with the exception of *N*_*e*_ associated with the bottleneck time prior. *N*_*e*_ parameters are subsequently referred to as *N*_*e(contemporary)*_, *N*_*e(bottleneck)*_, *N*_*e(historicA)*_, *N*_*e(historicB)*_, with the latter two being the *N*_*e*_ parameters associated with identical but independent time priors. Microsatellite *μ* was defined as the generalized stepwise mutation model (Estoup et al. 2002) with a mean rate uniformly distributed between 1.00 × 10^−5^ and 1.00 × 10^−3^ substitutions/generation. Although the lower bound on this prior distribution is lower than that generally assumed for microsatellites, previous studies have indicated a reduced molecular evolutionary rate in whales as compared with that usually observed in mammals (Alter and Palumbi 2009). For mitochondrial sequences, the employed model of evolution determined through model testing in MEGA 5 (Tamura et al. 2011) was HKY + I (0.5) + G (0.05). This substitution rate was uniformly distributed between 1 × 10^−7^ and 1 × 10^−8^ substitutions/site/generation.

Sensitivity to prior assumptions in ABC inference has previously been acknowledged by other authors (Chan et al. 2006; Hoffman et al. 2011). To explore the influence of prior assumptions about *N*_*e*_ on posterior estimates, additional simulations were performed incorporating a range of prior bounds on *N*_*e*_. Subsequent simulations were performed in which the *N*_*e*_ parameters originally defined between one and 20,000 were confined to an upper bound of 10,000 (a biologically reasonable upper bound given estimates of ancestral census size), or extended to 50,000, and then to 100,000 in final simulations. Although these latter prior bounds could be interpreted as overly generous, Roman and Palumbi (2003) reported considerably larger *N*_*e*_ estimates for several whale species than is generally discussed for *B. mysticetus*. Comparative analysis of all sets of simulations allowed for a diagnosis of how the availability of biological information about *N*_*e*_ (and prior assumptions) influences posterior inference.

By ranking simulated summary statistics in relation to the observed following Cornuet et al. (2010), preliminary simulations showed that, while simulated microsatellite data fit the observed data reasonably well, simulated mitochondrial data were a poor fit for the observed data. This is consistent with a previous study of Antarctic fur seals by Hoffman et al. (2011), and indicated that the mitochondrial data did not contain resolution for the recent time frames being investigated in this analysis. Because of this, simulations exploring ranges of *N*_*e*_ assumptions over relatively recent times focused on the microsatellite portion of the dataset. The mitochondrial data were instead analyzed using the alternative methodologies described above, which were not confined to specific time periods.

For all ABC analyses, 1 million simulated datasets were generated for each demographic model. Heterozygosity and the mean number of alleles were then computed as summary statistics for the observed and simulated datasets. These parameters were specifically selected because they are known to be influenced by changes in effective population size (Luikart et al. 1998). Model comparisons implemented the local linear regression method introduced by Beaumont et al. (2002). Type I and II error rates in model selection were calculated by simulating 500 datasets under the parameters of each model and assuming the given model was the correct model (Bertorelle et al. (2010) has discussed the advantage of using pseudo-observed datasets for evaluating the accuracy of analyses). From the best supported model, 10,000 datasets with the smallest Euclidean distances from the observed were retained to build posterior parameter distributions, which were smooth weighted using the Locfit function within R version 2.9.1 (R Development Team 2005).

## Results

For the microsatellite dataset, moderate to high levels of genetic variability were found, with each locus yielding between six and 28 alleles (mean = 15.7, Table 2) and expected heterozygosity ranging from 0.566 to 0.938 (mean = 0.808). Weakly significant deviations from Hardy–Weinberg equilibrium were detected at four loci (Table 2), but none of these remained significant following table-wide Bonferroni correction for multiple statistical tests (Hochberg 1988). Similarly, no evidence was found for null alleles being present at high frequencies in any of the loci. Tests for linkage disequilibrium yielded 19 significant *P*-values (*P* < 0.05) of 231 pairwise comparisons, only one of which remained significant following Bonferroni correction (loci Bmy10 and Bmy26). From the concatenated three gene mitochondrial dataset including 168 individuals, 86 haplotypes were identified (Appendix 1). Nucleotide diversity was estimated at 0.004 ± 0.002, and mean number of pairwise differences was 9.75 ± 4.49. Haplotypes were derived from 102 variable positions, including 95 transition, six transversions, and one indel in the HVR1 gene region.

### Classical sequence-based demographic tests

Both Tajima's *D* and Fu's *F*_*S*_ were negative and statistically significant (Tajima's *D* = −1.4, *P* < 0.05; Fu's *F*_*S =*_ −24.3, *P* < 0.0001). The mismatch distribution was unimodal with a raggedness index of 0.003 (the probability that simulated raggedness was greater than or equal to the observed raggedness was 0.89). Average *μ* for the concatenated mitochondrial gene regions was estimated at 1.18%/million years, τ was estimated at 4.34. Following a generation time of 52 years (Taylor et al. 2007), the estimated time to the demographic expansion was calculated at 75,296 ybp.

### Extended Bayesian skyline plot

**Figure 3:**
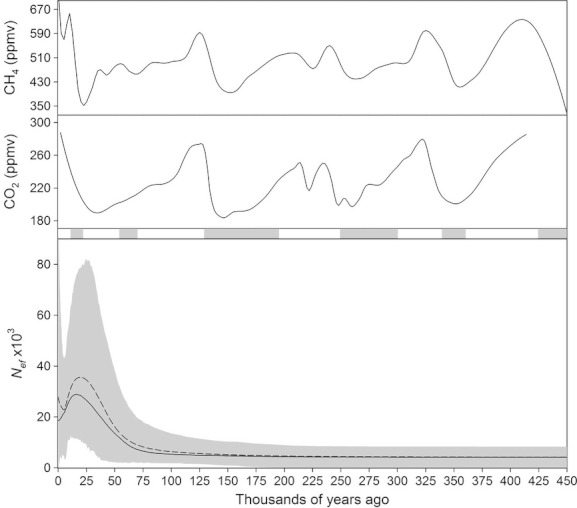
Extended Bayesian skyline reconstructions of *N*_*ef*_, timing of six past glacials (Rohling et al. 1998; gray bars denote glacials), CO2 (Petit et al. 1999), and CH4 (Loulergue et al. 2008) atmospheric concentrations plotted over time. For the demographic reconstruction, the gray area denotes the 95 highest posterior densities for the estimates, the hashed line represents the mean, and the solid line represents the median estimates.

Survey of effective sampling of values in TRACER disclosed values of greater than 200 for all parameters, indicating sufficiently deep sampling (Drummond and Rambaut 2007). Posterior estimates of mutation rates for HVRI, Cytb, and ND1 were calculated at 2.52%, 0.58%, and 0.49%/my, respectively. Reconstructions indicated a demographic history involving a few major episodes of population increase and decline, which were corroborated through independent replicates of the analysis yielding the same results. The demographic reconstruction included an increase in *N*_*ef*_ estimated to have begun between 50,000 and 75,000 ybp that continued until about 15,000 ybp, which was followed by a subsequent population reduction (Fig. 3). From this reconstruction, current *N*_*ef*_ was estimated at a median value of 20,000; however, the corresponding 95% highest posterior densities during this period were large.

### Classical microsatellite-based bottleneck tests

Analysis of the microsatellite dataset within the program BOTTLENECK yielded virtually identical results regardless of whether the full dataset was used or the analysis was restricted to the 18 loci in HWE (Table 3). There was also strong consistency among *P*-values obtained from the sign, standardized differences, and Wilcoxon tests. However, the results were highly dependent on the mutational model specified, with a significant excess of heterozygosity being detected under the IAM, but a significant deficiency of heterozygosity being found under the SMM. Similarly, the intermediate TPM models indicated a significant excess of heterozygosity when strongly influenced by the IAM (e.g., the TPM70 model) and a significant deficiency of heterozygosity when mutations were predominantly SMM (e.g., the TPM99 and TPM95 models).

**Table 3:** The number of loci exhibiting heterozygosity excess and test probabilities obtained using a range of mutational models (see Methods for details) within the program Bottleneck. Results are presented for separate analyses based on (a) the entire dataset; and (b) only the 18 loci that did not deviate significantly from HWE prior to correction for multiple tests. The mode test revealed normal L-shaped distributions under all of the scenarios tested. *P*-values significant at *α* < 0.05 without correction for multiple statistical tests are highlighted in bold

Mutational model	No. of loci with heterozygosity excess	No. of loci with heterozygosity deficiency	Sign test *P*-value	Standardized differences test *P*-value	Wilcoxon test *P*-value
All 22 loci:					
IAM	22	0	**<0.0001**	**<0.0001**	**<0.0001**
TPM70	18	4	**0.02129**	**0.01204**	**0.00528**
TPM90	12	10	0.43945	0.38970	0.87360
TPM95	8	14	**0.03066**	**0.02695**	0.13749
TPM99	4	16	**0.00279**	**<0.0001**	**0.00192**
SMM	5	17	**0.0006**	**<0.0001**	**0.0003**
18 loci in HWE:					
IAM	22	0	**<0.0001**	**<0.0001**	**<0.0001**
TPM70	18	4	**0.02129**	**0.01204**	**0.00528**
TPM90	12	10	0.43945	0.38970	0.87360
TPM95	8	14	**0.03066**	**0.02695**	0.13749
TPM99	4	16	**0.00279**	**<0.0001**	**0.00192**
SMM	5	17	**0.0006**	**<0.0001**	**0.0003**

### Approximate Bayesian computation

Comparison of demographic models indicated the model not enforcing a recent *N*_*e*_ reduction produced simulated datasets yielding summary statistics most similar to the observed. This model received a posterior probability of 0.85, while the model enforcing a genetic bottleneck yielded a posterior probability of 0.15. Type I and Type II error rates for the selection of the best supported model were 0.28 and 0.3, respectively. The selected model was described by an ancient *N*_*e*_ with a median value of 8980 (95% CI 1700–18,600; very similar values were obtained for both *N*_*e(historicA)*_ and *N*_*e(historicB)*_; Table 4). No resolution was recovered for posterior estimates for the times associated with these *N*_*e*_ estimates (data not shown because posterior distributions were flat). Similarly, posterior distributions associated with the whaling period time prior, its associated *N*_*e(bottleneck)*_, and *N*_*e(contemporary)*_ were all uninformative.

**Table 4:** Point estimates and 95% credibility intervals for all *N*_*e*_ and *μ* obtained through simulations evoking different prior assumptions on *N*_*e*_. ^*^ = because the posterior distributions for these parameters were mostly flat (Fig. 2) point estimates for these parameters are weak estimates

Parameter	Mean	Median	5%	95%
*N*_*e*_ (1–10,000)				
*N*_e(contemporary)_^*^	5.33 × 10^3^	5.37 × 10^3^	9.53 × 10^2^	9.54 × 10^3^
*N*_e(bottleneck)_^*^	6.22 × 10^3^	6.38 × 10^3^	2.13 × 10^3^	9.66 × 10^3^
*N*_*e(historicA)*_	5.71 × 10^3^	5.77 × 10^3^	1.38 × 10^3^	9.50 × 10^3^
*N*_*e(historicB)*_	5.74 × 10^3^	5.82 × 10^3^	1.45 × 10^3^	9.56 × 10^3^
*μ*	6.80 × 10^−4^	6.97 × 10^−4^	3.24 × 10^−4^	9.70 × 10^−4^
*N*_*e*_ (1–20,000)				
*N*_e(contemporary)_^*^	1.04 × 10^4^	1.05 × 10^3^	1.41 × 10^3^	1.90 × 10^4^
*N*_e(bottleneck)_^*^	1.17 × 10^4^	1.20 × 10^3^	3.07 × 10^3^	1.93 × 10^4^
*N*_*e(historicA)*_	9.60 × 10^3^	8.98 × 10^3^	1.70 × 10^3^	1.86 × 10^4^
*N*_*e(historicB)*_	9.71 × 10^3^	9.12 × 10^3^	2.03 × 10^3^	1.87 × 10^4^
*μ*	5.41 × 10^−4^	5.24 × 10^−4^	1.74 × 10^−4^	9.46 × 10^−4^
*N*_*e*_ (1-50,000)				
*N*_e(contemporary)_^*^	2.52 × 10^4^	2.50 × 10^4^	2.93 × 10^3^	4.74 × 10^4^
*N*_e(bottleneck)_^*^	2.74 × 10^4^	2.79 × 10^4^	5.50 × 10^3^	4.77 × 10^4^
*N*_*e(historicA)*_	2.01 × 10^4^	1.69 × 10^4^	3.01 × 10^3^	4.57 × 10^4^
*N*_*e(historicB)*_	2.04 × 10^4^	1.74 × 10^4^	3.01 × 10^3^	4.60 × 10^4^
*μ*	4.10 × 10^−4^	3.54 × 10^−4^	7.29 × 10^−5^	9.09 × 10^−4^
*N*_*e*_ (1–100,000)				
*N*_e(contemporary)_^*^	5.10 × 10^4^	5.08 × 10^4^	5.73 × 10^3^	9.53 × 10^4^
*N*_e(bottleneck)_^*^	5.32 × 10^4^	5.39 × 10^4^	8.84 × 10^3^	9.55 × 10^4^
*N*_*e(historicA)*_	3.62 × 10^4^	2.78 × 10^4^	3.66 × 10^3^	9.17 × 10^4^
*N*_*e(historicB)*_	3.60 × 10^4^	2.79 × 10^4^	3.64 × 10^3^	9.08 × 10^4^
*μ*	3.51 × 10^−4^	2.77 × 10^−4^	3.80 × 10^−5^	8.93 × 10^−4^

**Figure 4:**
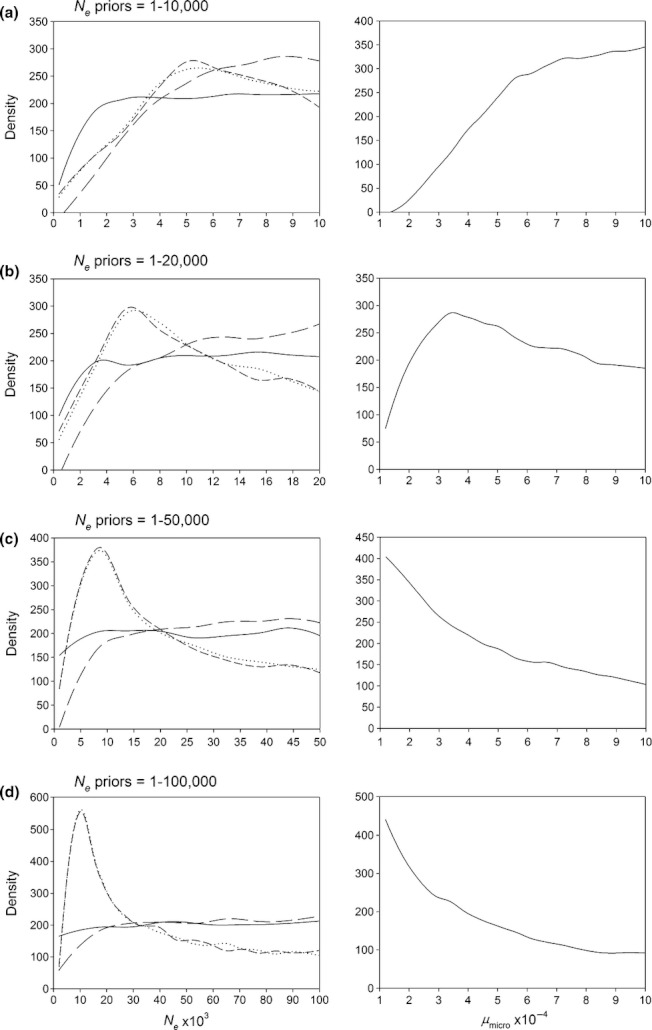
Plots of the posterior estimates of *N*_*e*_ values and *μ* under various prior assumptions on *N*_*e*_. There were five *N*_*e*_ priors with associated time priors defined for analysis (see Materials and Methods for details). The posterior estimates for these parameters are depicted in panels of the left column and *N*_*e(contempory)*_ = black line, *N*_*e(bottleneck)*_ = long dashes, *N*_*e(historicA)*_ = short dashes, and *N*_*e(historicB)*_ = dotted. The panels of the right column depict the posterior estimates for average *μ*, and the organization of panels within rows (a–d) corresponds to the prior bounds on *N*_*e*_ values that were assumed.

To explore sensitivity to priors on *N*_*e*_ and *μ*, we conducted a series of supplementary ABC simulations (see Materials and Methods for details). In all cases, the same demographic model was supported as in the initial simulations. However, broader prior assumptions on *N*_*e*_ yielded larger posterior estimates for ancestral *N*_*e*_ coupled with smaller posterior estimates for average *μ*. The posterior distributions of time and *N*_*e*_ parameters from all simulations are available as joint plots in Figure 4 and are listed in Table 4.

## Discussion

Demographic studies are inherently complicated by the fact that the histories of many species consist of multiple, temporally stratified events of different magnitudes, which are estimated from a single source of variation (i.e., the genome). This study used 22 highly polymorphic microsatellite loci and 2494 base pairs of mtDNA to explore historic demographic phenomena over multiple timescales and using a variety of contrasting but complimentary approaches. We recovered signal of ancient demographic change over time, but no signature of recent anthropogenic exploitation. The observed demographic patterns probably reflect the unique life-history characteristics of *B. mysticetus*, as discussed in detail below.

### Evidence for historic population expansion

Analyses of the mitochondrial dataset using various statistical approaches yielded consistent results. Tajima's *D* and Fu's *F*_*S*_ were found to be significantly negative, both of which are indicative of population expansion. Furthermore, the observed unimodal mismatch distribution not only corroborated these results but also provided an estimated time of expansion of roughly 75,000 ybp. This estimate was supported by results of the EBSP in which a similarly timed population expansion was reconstructed. This confirms the postulated population expansion detected by Rooney et al. (1999), but contradicts their hypothesis that the M'Clintock Channel Sea Ice Plug formation roughly 8500 years ago was implicated in the timing of the expansion. A subsequent study by Rooney et al. (2001) provided an alternative analysis and hypothesis for population expansion. These authors constructed lineage through time plots, and estimated coalescence time of *B. mysticetus*. The coalescence time was estimated to approximately 270,000 ybp and it was assumed that the coalescent event was directly followed by population expansion that is only now beginning to wane (from the continual estimated increase in number of lineages through time). In this study, we confirm the previous reports of a historic population expansion, but also provide a more robust estimation for the nature and timing of this expansion.

The EBSP, although providing estimates of *N*_*ef*_ that are likely inflated (potential bases for inflated effective population sizes are discussed by Kuhner et al. (1998) and Ho et al. (2005)), was valuable for providing improved resolution on the demographic history of *N*_*ef*_, while also providing further support for a population expansion, having begun about 75,000 years ago, and peaking around 25,000 years ago. This historic event is putatively the source of the population expansion signal obtained from multiple analyses. It should be noted that influence of unknown immigrations from other stocks could contribute a false signal for population expansion (e.g., Hutchinson et al. 2003). However, this seems relatively unlikely because a consistent signal for population expansion was found using a variety of analytical approaches, each of which considers different aspects of the data. In addition, the EBSP also described a subsequent population reduction, estimated to have taken place over the past 15,000 years. This postulated reduction pre-dates the period of known anthropogenic reduction in the 19th and 20th centuries. Although a tentative explanation for this pattern could be anthropogenic depletion by pre-historic humans, all available data indicate that whaling was not practiced by natives until around 2000 years ago (Stoker and Krupnik 1993). Therefore, it is more likely that natural biological cycles associated with carrying capacity and/or environmental change could be implicated. Fortunately, accurate climatologic reconstructions over the past several 1000 years have been corroborated using multiple data types through multiple studies (Rohling et al. 1998, 2009; Siddall et al. 2003). The major population expansion recovered by EBSP falls directly within the second previous glacial (70–65,000 years ago; Rohling et al. 1998), and the subsequent population decline (estimated to have begun 15,000 years ago) coincides with the warming and sea level rises starting at the end of the last glacial (15,000 years ago; Severinghaus and Brook 1999 and references therein). Plotted within Figure 3 are estimated historic levels of atmospheric CO_2_ (Petit et al. 1999) and CH_4_ (Loulergue et al. 2008), measured from Antarctic ice cores. Comparisons of these plots with the ESBP reveal that the period of population expansion broadly coincides with a time interval of low gas concentrations, while the timing of the population decline markedly corresponds to increased atmospheric gas concentrations (it should be recognized that the highest posterior density around population sizes as well as error that could be attributed to mutation rate and generation time attribute uncertainty to these qualitative comparisons). It is notable that although the fluctuations in gas concentrations are dynamic over the entire data range, demographic reconstructions are uninformative over deeper timescales. This observation is likely a reflection that more recent demographic events have eroded signal for more ancient phenomena. Trends observed between effective population size and climate change are most readily explained through their relationship with ecosystem carrying capacity and available habitat. This connection is particularly evident in this study, in which baleen filtering of planktonic communities is required to support large biomasses. As ocean productivity is largely determined by temperature (Behrenfeld et al. 2006), long-term climatic oscillations have likely shaped trends in the carrying capacity of the northern oceans. Behrenfeld and Falkowski (1997) estimated that the entire global ocean phytoplankton biomass is transferred through marine ecosystems (partly by grazing) every 2–6 days. The biomass turnover documented by these authors indicates the close connection between climate and effective population size of marine species. These climatologically directed ecosystem changes appear to be manifested in our reconstructions of *B. mysticetus* effective population size over time. Green-houses gases such as methane and carbon dioxide are suitable indicators for past climate, although related oceanographic factors (e.g., ice cover, meltwater, salinity, circulation) also likely contributed to environmental conditions, and hence bowhead whale historic demography in the Arctic.

### Evaluation of an anthropogenic bottleneck scenario

Initial bottleneck analysis applied the heterozygosity excess test under a range of mutation model definitions, finding a signal for a genetic bottleneck only under the IAM model. Given that this model is unrealistic for most “real” microsatellites (Di Rienzo et al. 1994) and that a TPM model comprising ∼5% IAM mutations is more likely (Piry et al. 1999), results from this analysis appear most consistent with the population having undergone a historic expansion. Moreover, a shift in the allele frequency distribution from an L-shaped distribution was not observed, suggesting that the population was not recently bottlenecked. Results of the ABC analysis, conducted as an alternative analytical bottleneck assessment, were generally in agreement with the classical bottleneck tests, yet provided greater demographic resolution. Given that the compared models only differed in the prior constraint on *N*_*e(bottleneck)*_, and that Type I and II error were both estimated at approximately 30%, results established that a population model involving a recent *N*_*e*_ reduction is not compatible with the observed distribution of genetic variation in *B. mysticetus*. In addition, the ABC analysis found no evidence for the population expansion circa 8500 ybp postulated by Rooney et al. (1999). The absence of these events in the recent demographic history is reflected in the posterior distributions of parameters. Similar *N*_*e*_ posteriors for *N*_*e(historicA)*_ and *N*_*e(historicB)*_ and flat distributions of time posteriors associated with these parameters indicate a lack of signal for shift in population size during this time frame (i.e., no population expansion 8500 ybp). Similarly, we found no clear signal for *N*_*e*_ during the past 300 years (*N*_*e(contemporary)*_ and *N*_*e(bottleneck)*_). The absence of a strong posterior estimate for *N*_*e*_ over recent times reflects that a wide range of *N*_*e*_ values over this time period in conjunction with estimated historic *N*_*e*_ values produce summary statistics similar to that observed in the BCB. In other words, any range of plausible population sizes over the past 300 years is not sufficient to drive genetic signal in bowhead whales. This is an important point, as it reflects the relationships among genetic signal, generation time, and *μ*, which are clearly manifested in this analysis because of the design of approximate Bayesian computation, which allows for the parameterization of models incorporating stratified temporal events.

**Figure 5:**
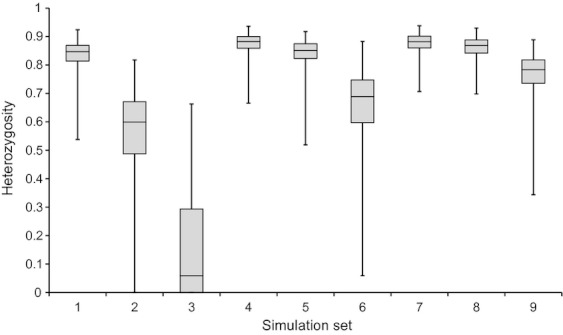
Box plots of heterozygosity values obtained through 1000 simulations for various combinations of *N*_*e(contemporary)*_, *N*_*e(historic)*_, and generations since population reduction. The combination of parameters labeled 1-9 on the horizontal axis were 1 = 10,000, 50, 5; 2 = 10,000, 50, 50; 3 = 10,000, 50, 500; 4 = 10,000, 500, 5, 5 = 10,000, 500; 50; 6 = 10,000, 500, 500, 7 = 10,000, 1000, 5; 8 = 10,000, 1000, 50; and 9 = 10,000, 1000, 500, respectively. Error bars indicate maximum and minimum observed values, gray boxes are 50th and 75th percentiles, and the median value is denoted as a black horizontal line. Simulation 7 represented the combination of parameter values most similar to that postulated for the BCB stock of bowhead whales.

The results of bottleneck analyses could be viewed as contrary to what would be expected given what is known about stock depletion through parts of the 19^th^ and 20^th^ centuries. The period of unregulated whaling, which ensued for nearly 70 years, resulted in greater than 90% population depletion. However, this time period measured in terms of *B. mysticetus* generations is only 1.3 generations. Thus, it seems that the perceived brief duration of the bottleneck has prevented the loss of genetic variation in *B. mysticetus*. This hypothesis is supported through theoretical work by Allendorf (1986), who through simulation showed that the reduction in heterozygosity resulting from population bottlenecks is largely determined by bottleneck duration. A few empirical studies have also observed a buffering effect against bottlenecks by long generation time in certain species (Dinerstein and McCracken 1990; Hailer et al. 2006; Lippé et al. 2006). By comparison, demographic studies investigating population depletion in the gray whale (Alter et al. 2007, 2012) have indicated population size reductions; however, the influence of human disturbances to the patterns is not clear. To illustrate this point, we carried out a set of *post hoc* simulations using SPAms (Parreira et al. 2009). The major parameters of the simulations involved *μ* (assumed as 1 × 10^−4^), *N*_*e(ancestral)*_, *N*_*e(contemporary)*_, and generations since the change in population size. Multiple sets of simulations were conducted to encompass a range of combinations of the latter three parameters. Figure 5 shows that the set of simulations most similar to that characterizing *B. mysticetus* resulted in no loss of heterozygosity. In fact, much smaller contemporary *N*_*e*_ coupled with bottlenecks of greater duration would be required to appreciably reduce heterozygosity. Apart from this simple relationship between generation time and bottleneck duration that has directed a lack of genetic signal in bowhead whales, an additional consideration is that contemporary sampling includes individuals that were born both before and after the population reduction (Givens et al. 2010). Because individuals born during or before the depletion are still living and potentially breeding provides additional buffering to bottleneck effects and influences the ability to detect genetic signature for the depletion.

An additional observation of the ABC analysis was the influence of prior assumptions on posterior estimations, and hence biological interpretations. This observation is not a characteristic unique to ABC, but to Bayesian methods in general. In this study, through a series of simulations that evoked differing degrees of constraint on *N*_*e*_ values, it was found that level of constraint applied was reflected in the posterior estimates. Specifically, tighter and lower prior assumptions on *N*_*e*_ yielded lower point estimates for this parameter. In addition, the inversely proportional relationship between *μ* and *N*_*e*_ was illustrated through observed posterior estimates obtained through a range of prior assumptions. In the case of *B. mysticetus*, or any species of management concern and with a tendency for small effective population sizes, assumptions (and uncertainty) on these parameters can directly influence the biological results upon which management decisions are made. As a priori information about *N*_*e*_ in bowhead whales and *μ* for whales are available, results obtained from some of the simulations can likely be excluded. For example, although posterior estimates for historic *N*_*e*_ (5700) stemming from simulations evoking a 1–10,000 *N*_*e*_ prior could be considered biologically plausible, a rather large value of *μ* was required, which is not likely given the generally low rates of molecular evolution previously observed in whales (Alter and Palumbi 2009). The true value of historic *N*_*e*_ likely lies within the estimates provided by the simulations with *N*_*e*_ priors of 1–20,000 and 1–50,000, which yielded mean estimates for historic *N*_*e*_ of about 10,000 and 20,000, respectively. However, the ability to further pinpoint historic *N*_*e*_ within this range would require a firmer assumption on *μ*.

### Practical implications

Our results have important implications for understanding the evolutionary basis of contemporary management practices. For *B. mysticetus*, an annual harvest quota for the Alaskan and Russian aboriginal hunt is provided by the International Whaling Commission (IWC) following the Aboriginal Whaling Management Procedure (AWMP). Following the AWMP, the existing *B. mysticetus* assessment is driven by the past catch series, the current estimate of abundance in absolute terms, the current rate of population increase, and other life-history parameters. The current population growth rate is estimated to be 3.5% (George and Zeh 2012). However, the long-term growth rate over the past century is dependent upon the size of the population at its nadir. Taken together and under the conventional population model assumption of compensatory dynamics (the larger the population, the lower the per-capita growth rate), there is an indication that the population was small at the end of the 19th century (D. Butterworth, pers. comm.). Despite abundance estimates not being available for this period, the results of this study suggest that the period of unregulated harvest was initiated during, or perhaps at the end of, a natural population reduction, which corresponds to climatic oscillations. Thus, the current population increase is likely a response to the compound effects of natural and anthropogenic occurrences.

The estimated population size prior to commercial whaling is another important component of the IWC's management strategy. In 1974, the IWC established that a species' original population size is to be used as an index for comparison of the estimated current population size to classify species into management categories and for setting harvest quotas (International Whaling Commission 1976). Woodby and Botkin (1993) reviewed the previous estimates of pre-whaling population size of the BCB population and developed a “simple recruitment model” to obtain a best estimate of the range of possible population sizes. These authors conclude that the estimated population size ranged from 10,400 to 23,000. Brandon and Wade (2006) suggest much the same range, but favor estimates near 14,000 using a Bayesian model averaging approach. However, they note “there is no [visual] evidence in the abundance estimates for a reduction in trend” suggesting that a value larger than 14,000 is likely (which is close to present abundance of ∼12,600; Koski et al. 2010). Brandon and Wade (2006) do not estimate the population nadir. These estimates agree closely with the historic *N*_*e*_ of 10,000–20,000 estimated from the simulations in this article following estimates that the mature proportion of the BCB population is about 40% (Bockstoce and Botkin 1983; Woodby and Botkin 1993; Koski et al. 2006). This gives further reassurance to the current strike limit established by the IWC as population estimates based on both genetic data and historic catch data (Woodby and Botkin 1993) are based on entirely different sets of assumptions.

### Conclusions

Important findings have emerged from this course of study pertaining to different aspects of demographic reconstruction. Demographic responses to both anthropogenic and natural environmental pressures are dictated by magnitude, duration, and species-specific life-history characteristics. For *B. mysticetus*, long generation time and dietary strategy are life-history characteristics that appear to be central to this species' demographic history. While long generation time served as a buffer to population bottleneck effects, putative long-term changes in Arctic carrying capacity drove a dynamic historic demography that is readily detected using a variety of statistical approaches.

## Appendix 1

Sample identifications and corresponding haplotypes for each mitochondrial gene region. Haplotype sequences are available at Genbank under accession numbers [numbers pending]. Lettering within each sample ID indicates collection locality, with B = Barrow, KK = Kaktovik, G = Gambell, S = Savoonga, N = Nuiqsut, H = Point Hope, WW = Wainwright.

**Table:**

GeneticID	HVR1_Haplotype	Cytb_haplotype	ND1_haplotype
00B2	BH42	Cytb_2	ND1_1
00B3	BH42	Cytb_40	ND1_1
00B4	BH5	Cytb_32	ND1_1
00B5	BH42	Cytb_1	ND1_12
00B6	BH42	Cytb_1	ND1_1
01B1	BH58	Cytb_5	ND1_2
01B10	BH4	Cytb_31	ND1_7
01B12	BH42	Cytb_1	ND1_1
01B14	BH64	Cytb_18	ND1_34
01B15	BH42	Cytb_2	ND1_1
01B17	BH5	Cytb_31	ND1_1
01B2	BH42	Cytb_1	ND1_31
01B3	BH46	Cytb_1	ND1_1
01B4	BH15	Cytb_31	ND1_1
01B6	BH31	Cytb_5	ND1_33
01B7	BH62	Cytb_1	ND1_1
01B8	BH5	Cytb_31	ND1_1
02B10	BH39	Cytb_6	ND1_1
02B12	BH42	Cytb_5	ND1_1
02B14	BH42	Cytb_5	ND1_1
02B15	BH1	Cytb_39	ND1_4
02B16	BH15	Cytb_31	ND1_1
02B17	BH34	Cytb_5	ND1_2
02B18	BH1	Cytb_39	ND1_4
02B19	BH42	Cytb_37	ND1_1
02B2	BH42	Cytb_1	ND1_4
02B20	BH48	Cytb_41	ND1_1
02B21	BH31	Cytb_5	ND1_33
02B22	BH58	Cytb_5	ND1_2
02B8	BH5	Cytb_32	ND1_1
02G2	BH31	Cytb_5	ND1_33
02KK1	BH23	Cytb_29	ND1_23
02KK2	BH15	Cytb_31	ND1_1
02S2_4	BH42	Cytb_43	ND1_1
02S3	BH64	Cytb_18	ND1_34
02S5	BH42	Cytb_5	ND1_1
03B12	BH19	Cytb_20	ND1_1
03B13	BH46	Cytb_1	ND1_1
03B14	BH5	Cytb_31	ND1_1
03B16	BH42	Cytb_43	ND1_1
03B2	BH42	Cytb_5	ND1_1
03KK1	BH42	Cytb_37	ND1_1
03KK2	BH42	Cytb_1	ND1_12
03S1	BH34	Cytb_1	ND1_1
03S2	BH42	Cytb_1	ND1_12
04B1	BH23	Cytb_31	ND1_1
04B10	BH23	Cytb_30	ND1_24
04B11	BH55	Cytb_16	ND1_2
04B12	BH31	Cytb_5	ND1_33
04B13	BH25	Cytb_34	ND1_1
04B14	BH66	Cytb_1	ND1_1
04B15	BH42	Cytb_1	ND1_1
04B16	BH10	Cytb_11	ND1_2
04B17	BH42	Cytb_1	ND1_1
04B18	BH13	Cytb_9	ND1_1
04B20	BH17	Cytb_20	ND1_1
04B3	BH5	Cytb_31	ND1_1
04B4	BH61	Cytb_42	ND1_1
04B5	BH32	Cytb_17	ND1_2
04B6	BH43	Cytb_39	ND1_4
04B7	BH7	Cytb_31	ND1_20
04B9	BH2	Cytb_12	ND1_2
04G3	BH28	Cytb_28	ND1_1
04KK1	BH46	Cytb_3	ND1_1
04KK2	BH42	Cytb_43	ND1_1
04KK3	BH33	Cytb_14	ND1_2
04N1	BH51	Cytb_20	ND1_3
04N2	BH20	Cytb_25	ND1_8
04N3	BH20	Cytb_20	ND1_1
05B1	BH61	Cytb_42	ND1_1
05B10	BH42	Cytb_1	ND1_26
05B11	BH43	Cytb_39	ND1_4
05B12	BH59	Cytb_5	ND1_2
05B13	BH23	Cytb_31	ND1_1
05B15	BH28	Cytb_34	ND1_1
05B16	BH53	Cytb_34	ND1_17
05B17	BH61	Cytb_42	ND1_25
05B18	BH24	Cytb_31	ND1_1
05B19	BH42	Cytb_36	ND1_1
05B2	BH20	Cytb_25	ND1_8
05B20	BH42	Cytb_2	ND1_1
05B21	BH46	Cytb_1	ND1_31
05B22	BH36	Cytb_16	ND1_2
05B23	BH11	Cytb_1	ND1_1
05B24	BH29	Cytb_34	ND1_1
05B25	BH7	Cytb_31	ND1_20
05B26	BH64	Cytb_18	ND1_20
05B27	BH41	Cytb_42	ND1_34
05B28	BH49	Cytb_44	ND1_1
05B3	BH7	Cytb_31	ND1_20
05B4	BH42	Cytb_36	ND1_1
05B5	BH36	Cytb_16	ND1_2
05B6	BH42	Cytb_1	ND1_4
05B7	BH64	Cytb_18	ND1_34
05B8	BH42	Cytb_1	ND1_1
05B9	BH23	Cytb_31	ND1_1
05BpB1	BH9	Cytb_20	ND1_4
05BpB12	BH19	Cytb_31	ND1_1
05BpB8	BH42	Cytb_4	ND1_1
05G1	BH42	Cytb_1	ND1_1
05G2	BH5	Cytb_31	ND1_1
05H1	BH23	Cytb_34	ND1_1
05KK1	BH42	Cytb_1	ND1_1
05KK3	BH27	Cytb_25	ND1_8
05S1	BH47	Cytb_5	ND1_2
05S3	BH5	Cytb_31	ND1_1
05S4	BH7	Cytb_31	ND1_20
05S5	BH42	Cytb_1	ND1_1
05S6	BH43	Cytb_39	ND1_4
05S7	BH42	Cytb_5	ND1_1
05WW1	BH41	Cytb_20	ND1_14
05WW2	BH20	Cytb_42	ND1_1
05WW3	BH32	Cytb_5	ND1_32
05WW4	BH23	Cytb_31	ND1_13
06B1	BH42	Cytb_7	ND1_1
06B2	BH3	Cytb_42	ND1_1
06B3	BH42	Cytb_43	ND1_1
95B1	BH20	Cytb_23	ND1_1
95B4	BH27	Cytb_25	ND1_8
95B8	BH42	Cytb_36	ND1_1
95B9	BH42	Cytb_5	ND1_1
96B10	BH1	Cytb_42	ND1_4
96B11	BH23	Cytb_31	ND1_1
96B12	BH45	Cytb_42	ND1_4
96B14	BH1	Cytb_39	ND1_4
96B15	BH35	Cytb_5	ND1_2
96B16	BH28	Cytb_34	ND1_1
96B17	BH29	Cytb_34	ND1_1
96B18	BH46	Cytb_1	ND1_1
96B19	BH38	Cytb_6	ND1_1
96B2	BH4	Cytb_31	ND1_1
96B20	BH20	Cytb_20	ND1_1
96B21	BH42	Cytb_1	ND1_25
96B22	BH61	Cytb_42	ND1_1
96B23	BH42	Cytb_43	ND1_2
96B4	BH19	Cytb_20	ND1_1
96B5	BH61	Cytb_42	ND1_25
96B6	BH58	Cytb_5	ND1_2
96B7	BH42	Cytb_37	ND1_1
96B8	BH23	Cytb_31	ND1_1
96B9	BH51	Cytb_20	ND1_3
97B10	BH42	Cytb_42	ND1_22
97B12	BH4	Cytb_31	ND1_7
97B17	BH46	Cytb_1	ND1_1
97B18	BH39	Cytb_6	ND1_1
97B19	BH2	Cytb_11	ND1_2
97B2	BH42	Cytb_1	ND1_1
97B20	BH23	Cytb_34	ND1_1
97B21	BH64	Cytb_18	ND1_34
97B22	BH10	Cytb_11	ND1_2
97B24	BH21	Cytb_24	ND1_30
97B25	BH5	Cytb_31	ND1_29
97B26	BH23	Cytb_31	ND1_1
97B27	BH5	Cytb_31	ND1_1
97B28	BH42	Cytb_1	ND1_1
97B29	BH2	Cytb_11	ND1_2
97B3	BH18	Cytb_20	ND1_16
97B30	BH4	Cytb_31	ND1_7
97B31	BH42	Cytb_1	ND1_1
97B4	BH32	Cytb_5	ND1_32
98B24	BH42	Cytb_1	ND1_1
99B15	BH61	Cytb_42	ND1_1
99B17	BH5	Cytb_31	ND1_29
99B2	BH14	Cytb_1	ND1_31
99B21	BH23	Cytb_41	ND1_4
99B23	BH50	Cytb_20	ND1_3
99B3	BH23	Cytb_34	ND1_1
99B7	BH43	Cytb_39	ND1_4
